# Association between Subcortical Vascular Lesion Location and Cognition: A Voxel-Based and Tract-Based Lesion-Symptom Mapping Study. The SMART-MR Study

**DOI:** 10.1371/journal.pone.0060541

**Published:** 2013-04-08

**Authors:** J. Matthijs Biesbroek, Hugo J. Kuijf, Yolanda van der Graaf, Koen L. Vincken, Albert Postma, Willem P. T. M. Mali, Geert J. Biessels, Mirjam I. Geerlings, on behalf of the SMART Study Group

**Affiliations:** 1 Department of Neurology, Rudolf Magnus Institute of Neuroscience, University Medical Center Utrecht, Utrecht, The Netherlands; 2 Image Sciences Institute, University Medical Center Utrecht, Utrecht, The Netherlands; 3 Julius Center for Health Sciences and Primary Care, University Medical Center Utrecht, Utrecht, The Netherlands; 4 Experimental Psychology, Helmholtz Institute, Utrecht University, Utrecht, The Netherlands; 5 Department of Radiology, University Medical Center Utrecht, Utrecht, The Netherlands; INRCA, Italy

## Abstract

**Introduction:**

Lacunar lesions (LLs) and white matter lesions (WMLs) affect cognition. We assessed whether lesions located in specific white matter tracts were associated with cognitive performance taking into account total lesion burden.

**Methods:**

Within the Second Manifestations of ARTerial disease Magnetic Resonance (SMART-MR) study, cross-sectional analyses were performed on 516 patients with manifest arterial disease. We applied an assumption-free voxel-based lesion-symptom mapping approach to investigate the relation between LL and WML locations on 1.5 Tesla brain MRI and compound scores of executive functioning, memory and processing speed. Secondly, a multivariable linear regression model was used to relate the regional volume of LLs and WMLs within specific white matter tracts to cognitive functioning.

**Results:**

Voxel-based lesion-symptom mapping identified several clusters of voxels with a significant correlation between WMLs and executive functioning, mostly located within the superior longitudinal fasciculus and anterior thalamic radiation. In the multivariable linear regression model, a statistically significant association was found between regional LL volume within the superior longitudinal fasciculus and anterior thalamic radiation and executive functioning after adjustment for total LL and WML burden.

**Conclusion:**

These findings identify the superior longitudinal fasciculus and anterior thalamic radiation as key anatomical structures in executive functioning and emphasize the role of strategically located vascular lesions in vascular cognitive impairment.

## Introduction

Cognitive impairment is a common and disabling condition in older people [1]. Vascular lesions including subclinical white matter lesions (WMLs) and lacunes can cause or contribute to cognitive dysfunction [2].

A high degree of inter-individual variation exists regarding the relation between WMLs and lacunes and cognition. Both total lesion burden and lesion location appear to be relevant. An association has previously been demonstrated between cognitive functioning and lesion volume for lacunar and non-lacunar infarcts [3]–[6] and WMLs [7], [8]. In addition to lesion volume, the location of these lesions is an important factor in explaining variance in cognitive performance [9]. Known strategic locations include the thalamus, caudate nuclei, capsular genu and left angular gyrus. Infarcts in these locations are associated with poststroke dementia [2]. However, strategic locations may also include specific white matter tracts and lesions within these tracts might cause task-specific cognitive decline. Recently, a study of patients with CADASIL (Cerebral Autosomal Dominant Arteriopathy with Subcortical Infarcts and Leukoencephalopathy) demonstrated that strategically located lesions in white matter tracts had a stronger correlation with performance than total lesion volume [10]. In these patients, regional volumes of lacunar lesions (LLs) and WMLs located in the anterior thalamic radiation and forceps minor predicted performance in processing speed tasks, whereas there was no independent contribution of the global volume of ischemic lesions. In a cohort of patients with manifest arterial disease (SMART cohort) we previously demonstrated that periventricular WML volume and LLs in the semioval center were associated with poor executive functioning after adjusting for co-occuring infarcts and total WML volume [11]. These results suggest a strategic role of periventricular white matter in executive functioning. It might be reasoned that the impact of WMLs and LLs on executive functioning is likely to be caused by the disruption of strategic white matter tracts.

We aimed to investigate the relationship between the location of WMLs and LLs and cognitive functioning in a large cohort of patients with manifest arterial disease, using assumption-free voxel-based lesion-symptom mapping (VLSM). Secondly, using a region of interest-based analysis, we assessed whether lesion volume in strategic white matter tracts was associated with cognitive functioning after controlling for total lesion burden.

## Methods

### Ethics

The SMART-MR study was approved by the Medical Research Ethics Committee of the University Medical Center Utrecht and written informed consent was obtained from all participants.

### Subjects

The Second Manifestations of ARTerial disease Magnetic Resonance (SMART-MR) study is a prospective cohort study aimed at investigating brain changes on MRI in 1309 independently living patients with symptomatic atherosclerotic disease [12]. In brief, between May 2001 and December 2005, all patients newly referred to the University Medical Center Utrecht with manifest coronary artery disease, cerebrovascular disease, peripheral arterial disease or an abdominal aortic aneurysm, and without MR contraindications, were invited to participate. Between January 2006 and May 2009, all participants still alive were invited for follow-up measurements, including MRI of the brain and neuropsychological testing. In total, 754 of the surviving patients in the SMART-MR cohort (61% of n = 1238) gave written informed consent and participated at follow-up; 466 persons (38%) refused or did not respond, and 18 persons (1%) were lost to follow-up. Because cognitive functioning was assessed more extensively at follow-up than at baseline, only data from the follow-up assessment was used for the present study, which thus has a cross-sectional design.

Baseline characteristics of all patients included in the SMART-MR baseline and follow-up study are extensively described elsewhere [13]. Compared with the total SMART-MR population, patients with follow-up measurements were younger at baseline, less often had hypertension and diabetes and had fewer white matter lesions. Mean interval between measurements was 3.9 years (SD 0.4; range 3.0–5.8 years).

### Study Sample

Of the 754 patients included in the SMART-MR follow-up study, data on cognition were missing in 70 patients (no cognition tests owing to logistical problems: 18; incomplete test data owing to severe cognitive or behavioural impairment or visual or hearing handicaps: 52). Of the remaining patients, an adequate MRI scan was missing in 53 patients (no MRI: 37; motion or artefacts: 16). Registration of the MRI scan to standard space was unsuccessful in 39 patients (mostly because of atrophy and motion artefacts. Because the current study specifically aimed to assess the impact of small vessel disease (i.e. lacunes and WMLs) on cognition, 76 patients with territorial infarcts were excluded. This resulted in an analytical sample of 516 patients.

### MRI Protocol

MR investigations were performed on a 1.5 T whole-body system (Gyroscan ACS-NT, Philips Medical Systems, Best, The Netherlands). The protocol consisted of a transversal T1- weighted gradient-echo (GE) sequence (repetition time (TR)/echo time (TE): 235/2 ms), a transversal T2-weighted turbo spin-echo sequence (TR/TE: 2200/11 ms and 2200/100 ms), a transversal T2-weighted fluid-attenuated inversion recovery (FLAIR) sequence (TR/TE/inversion time (TI): 6000/100/2000 ms) and a transversal inversion recovery (IR) sequence (TR/TE/TI: 2900/22/410 ms) (field of view 2303230 mm; matrix size, 1803256; slice thickness, 4.0 mm; no gap, 38 slices).

### Brain Segmentation

We used the T1-weighted, IR, and FLAIR sequences for brain segmentation with a previously established method [14], [15]. The segmentation program identified cortical grey matter, white matter, sulcal and ventricular cerebrospinal fluid (CSF), and lesions. Results of the segmentation analysis were visually checked for the presence of infarcts and adapted if necessary to distinguish between WML and infarct volumes. Total brain volume was calculated by summing grey- and white-matter volumes and, if present, WML and infarct volumes. All volumes cranial to the foramen magnum were included. As a result, the total brain volume included the cerebrum, brainstem and cerebellum. Total intracranial volume (ICV) was calculated by summing total brain volume and sulcal and ventricular CSF volumes. Brain parenchymal fraction was calculated by dividing total brain volume by ICV and was expressed as a percentage of ICV.

The whole brain was visually searched for infarcts by two trained investigators and a neuroradiologist. Raters were blind to patient history and diagnosis. Discrepancies in rating were re-evaluated in a consensus meeting. Infarcts were defined as focal hyperintensities on T2-weighted images of at least 3 mm in diameter. Hyperintensities located in the white matter also had to be hypointense on T1-weighted and FLAIR images in order to distinguish them from WML. Dilated perivascular spaces were distinguished from infarcts based on their location (along perforating or medullary arteries, often bilateral and symmetrical, usually in the lower third of the basal ganglia or centrum semiovale), shape (round/oval) and absence of gliosis. Location, flow territory and type were scored for every infarct. Brain infarcts were categorised as cortical infarcts, lacunar lesions (LLs), and large subcortical infarcts. Large subcortical infarcts were >15 mm in size and were not confluent with cortical infarcts. LLs were defined as infarcts of 3–15 mm in diameter and located in the frontal, parietal, temporal and occipital lobe, corona radiata, internal capsule, semioval center, thalamus, basal ganglia, brain stem or cerebellar white matter.

### Neuropsychological Assessment

Memory, executive functioning, and processing speed and attention were assessed. For each of these three cognitive domains, composite z-scores were computed by averaging the z-scores of all subtests per domain [11]. Verbal memory was assessed with five consecutive trials of the 15-word learning test (a modification of the Rey Auditory Verbal Learning test [16]). It included both immediate and delayed recall scores. Non-verbal memory was assessed with the delayed recall of the Rey-Osterrieth Complex Figure test [17]. Executive functioning was measured using three tests. The visual elevator test (subtest of the Test of Everyday Attention [18]) is a timed test of 10 trials that measures mental flexibility and shifting of attention. The Brixton Spatial Anticipation test [19] was used to assess the capacity to discover logical rules and mental inhibition and flexibility. The total number of errors made was scored. The verbal fluency test (letter A, 1 min time frame) was used to assess mental flexibility and employment of strategies. Before calculating the z-scores, the scores of the Visual Elevator test and Brixton Spatial Anticipation test were multiplied by −1, so that lower scores represented poorer performance. Attention was measured with the Digit Span Forward and Digit Span Backward. In total, there were 16 trials, and the maximum of correct trials was scored. The Symbol substitution test (subtest from the Wechsler Adult Intelligence Scale) was administered to measure processing speed, and the number of correct responses during a 2 min time frame was scored. Educational level was divided into eight categories, graded from primary school to academic degree, according to the Dutch educational system. Intelligence was assessed using the validated Dutch Adult Reading Test (DART) [20].

### Generation of Lesion Maps

Registration of T1 images to a 2 mm MNI-152 (Montreal Neurological Institute) template [21], [22] was performed using elastix [23], with a linear registration followed by a non-linear registration [24]. For registration to standard space, a lesion masking approach was applied to enhance registration quality [25]. Lesion masks were created by subtracting WML and infarct lesion maps from brain masks. Visual checks of the results of the registration process were performed for all patients. After quality control of T1 images in MNI space, the warp fields were used to co-register the corresponding WML and infarct maps to the 2-mm MNI template. Total LL volume and WML volume after registration to MNI space were calculated for all patients.

### Statistical Analysis

We performed two separate analyses. First, we performed assumption-free voxel-based lesion-symptom mapping. Voxel-based lesion mapping can be used to determine relationships between behavioral measures and the location of brain injury, revealing the function of brain regions [26], [27]. One major advantage of this method over traditional approaches to lesion mapping is that instead of grouping of patients with or without lesions in one or more pre-defined areas of interest, it allows for assumption-free calculation of association at each voxel. Second, a region of interest-based analysis was performed using a white matter tract atlas [28]. Specific white matter tracts provided in the atlas were projected on the scans that were registered to standard space. Subsequently, WML and LL volumes with specific white matter tracts were calculated and associated with cognitive functioning using linear regression. Technical details regarding these analyses are provided below.

#### Assumption-free voxel-based lesion-symptom mapping

Analyses were done on the residuals of the composite z-scores for executive functioning, memory and processing speed after individualized correction for age, sex, level of education and DART using a linear regression model. Non-Parametric Mapping (most recent version, April 2010) was used to relate lesion location to cognitive performance [29] (settings: t-test, univariate analysis). Voxels affected in less than 5 patients were not considered for analysis. Correction for multiple testing was achieved by using a false detection rate (FDR) with q<0.05. Voxels reaching statistical significance were projected on the MNI template.

#### Region of interest-based analysis

**Figure 1:**
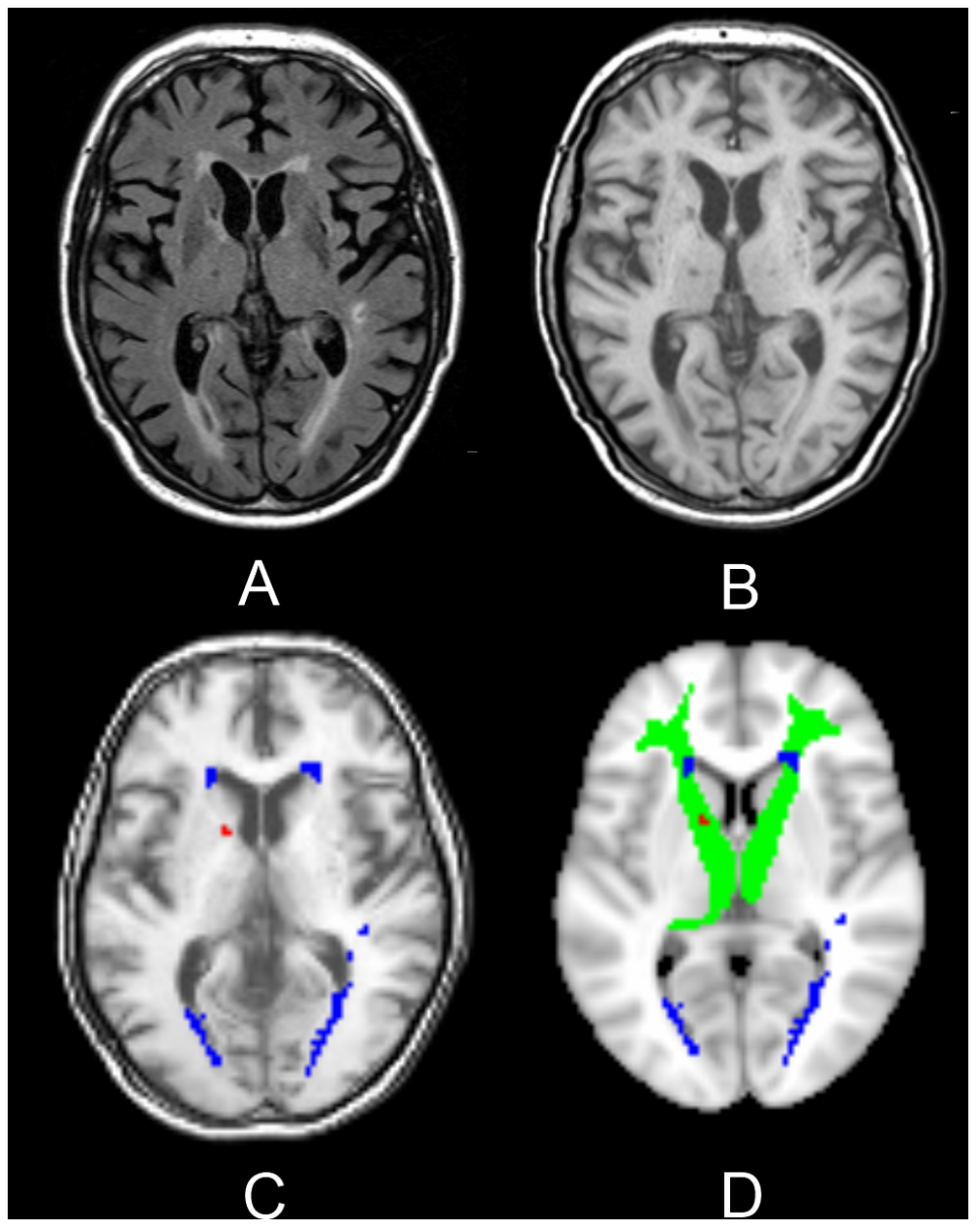
Segmentation of lesions within a specific white matter tract. The native T2 FLAIR (A) and T1 (B) sequences were used for segmentation of white matter lesions (WMLs) and lacunar lesions (LLs). The native T1 sequence was subsequently registered to MNI space followed by co-registration of the lesion maps using the same warp field. C: WMLs (blue) and LLs (red) projected on the registered T1 sequence. D: WMLs (blue) and LLs (red) projected on the MNI template. The anterior thalamic radiation is depicted in green. Regional lesion volumes within specific white matter tracts, in this case the anterior thalamic radiation, were associated with cognitive functioning using a linear regression model.

Information from the probabilistic white matter atlas (thresholded at 0.1) was used to create regions of interest for 10 major white matter tracts in MNI space [28]. These regions of interest were projected on the VLSM results and the amount of voxels with a statistically significant correlation within each white matter tract was visually assessed. White matter tracts containing more than 50 voxels or a single cluster of more than 5 voxels were selected for the subsequent region of interest-based analysis. The selected regions of interest were used to calculate WML and LL volumes within specific white matter tracts for every patient (an example is provided in figure 1). These regional lesion volumes were entered as independent variables in a step-wise linear regression before and after adding indicators of total lesion burden to the model with z-scores of cognitive functioning as the dependent variable.

## Results

Clinical and imaging characteristics of the study cohort are provided in table 1. Ninety-eight (19%) of the 516 included patients had one or more LLs. Nearly all patients (515 of 516) had WML in ≥1 voxel. The cognitive profile of the study cohort is provided in table 2.

**Table 1:** Characteristics of the study cohort.

Characteristics	Study cohort (n = 516)
Demographic characteristics	
Age, mean (SD)	56.7 (9.4)
Male, %	82
Inclusion diagnosis, % a	
Coronary artery disease	67
Cerebrovascular disease	15
Peripheral artery disease	18
Aortic aneurysm	5
Vascular risk factors, %	
Current smoker	31
Smoking history	48
Hypertension	45
Diabetes	11
Hypercholesterolaemia	40
Imaging characteristics	
Patients with LL, n (%)	98 (19)
Patients with LL in ATR, n (%)	40 (8)
Patients with LL in SLF, n (%)	25 (5)
Median LL volume, ml (range) b,c	0.17 (0.01–3.74)
Median WML volume, ml (range) b	0.9 (0–92)
Brain parenchymal fraction, mean (SD)	78.9 (2.8)

LL: lacunar lesion. ATR: anterior thalamic radiation. SLF: superior longitudinal fasciculus. WML: white matter lesion.

a Adds up to >100% because some patients had multiple diagnoses at inclusion.

b Data are based on volumes after registration to MNI space (normalised volumes).

c Among 98 patients with a LL.

**Table 2:** Cognitive profile of the study cohort.

Neuropsychological test	Mean (SD)	Range
15-word learning, immediate recall (no. correct, max 75)	40 (10)	5–63
15-word learning, delayed recall (no. correct, max 15)	8 (3)	0–15
Rey-Osterrieth Complex Figure, delayed recall (max 36)	21 (6)	0–35
Visual elevator test (seconds per switch)	4.8 (2.4)	2.0–31.1
Brixton Spatial Anticipation (no. of errors, max 54)	18 (7)	4–54
Verbal fluency, letter A (no. of words in one minute)	11 (5)	1–60
Digit span forward (no. correct, max 16)	8 (2)	2–15
Digit span backward (no. correct, max 14)	6 (2)	1–13
Symbol substitution test (no. correct in two minutes)	58 (15)	17–100

### Voxel-based Lesion-symptom Mapping

**Figure 2:**
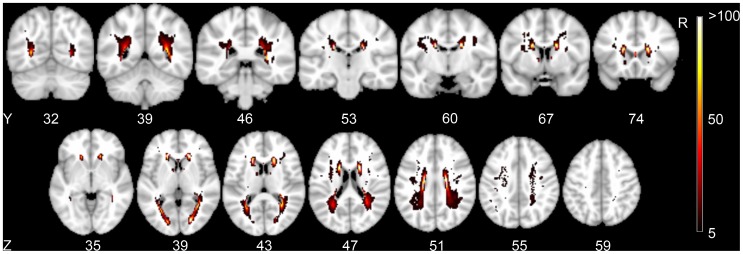
Frequency of white matter lesions. Voxels with white matter lesion (WML) in at least 5 patients are projected on a 2 mm MNI-152 template (Z and Y coordinates are provided). Bar indicates the number of patients with WML for each voxel.

**Figure 3:**
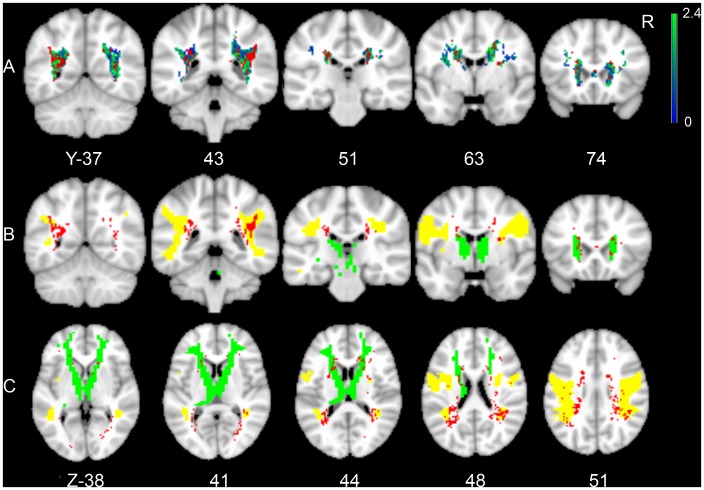
Voxel-based lesion-symptom mapping results. A: map of the correlation (t-statistic) between a lesion in each voxel and executive functioning. Voxels exceeding the false discovery rate threshold (q = 0.05 resulting in a threshold of t = 2.4) are rendered in red corresponding with a t-value >2.4, while non-significant voxels are rendered on a scale from blue to green corresponding with t-values ranging from 0 to 2.4. Results were adjusted for age, sex, level of education and performance on the Dutch Adult Reading Test. B and C: same analysis as A. Voxels exceeding the false discovery rate threshold and the superior longitudinal fasciculus (yellow) and anterior thalamic radiation (green) are depicted.

The distribution of WMLs is illustrated by the lesion prevalence map (figure 2); only voxels affected in at least 5 patients are shown. Assumption-free VLSM identified several clusters of voxels with a significant correlation between presence of WMLs and executive functioning after adjusting for age, sex, level of education and DART, and after correction for testing of multiple voxels (t-test range: −2.2 to 5.5; FDR (q = 0.05) threshold: t = 2.4). These significant clusters were located bilaterally in the posterior semioval center, mostly projecting on the superior longitudinal fasciculus and the anterior thalamic radiation, as identified on the probabilistic white matter atlas. The VLSM results are provided in figure 3. The correlation with executive functioning in these clusters did not remain statistically significant after additionally adjusting for total WML volume and LL volume. For memory and processing speed no significant clusters were found. VLSM could not be performed for LLs because no single voxel was affected in at least five patients.

### Region of Interest-based Linear Regression Model for Executive Functioning

In the next step, we assessed the association between WML and LL location and executive functioning using a region of interest-based linear regression model (table 3). Because in the VLSM analysis most significant clusters of voxels projected on the superior longitudinal fasciculus and anterior thalamic radiation, these tracts were used as regions of interest to calculate LL and WML volumes within these white matter tracts for every individual patient. In the linear regression model we first entered age, sex, level of education and DART; these variables explained most of the variance in executive functioning (R^2^ = 0.255, p = <0.001). Secondly, the presence of LLs (R^2^ increase: 0.004, p = 0.101) and total WML volume (i.e. throughout the brain) (R^2^ increase: 0.021, p = <0.001) were added to the model. Finally, regional LL and WML volumes within the superior longitudinal fasciculus and anterior thalamic radiation were added to the model. Adding regional LL volume within the superior longitudinal fasciculus (R^2^ increase: 0.007; B −1.20 (95%CI −2.24 to −0.16)) and anterior thalamic radiation (R^2^ increase: 0.006; B −3.12 (95% CI −6.13 to −0.11)) after adjustment for total vascular lesion burden and for age, sex, level of education and DART led to a statistically significant increase in explained variance. This was not the case when WML volume within the superior longitudinal fasciculus or anterior thalamic radiation was added to the model.

**Table 3:** Results of linear regression models with indicators of cerebral small vessel disease as variables and Z-scores of executive functioning as outcome.

Model	Independent variables	R^2^	*p*-value Δ R^2^	B (95% CI)
1	Age, sex, level of education, Dutch Adult Reading Test	0.255	<0.001	
2a	Model 1+ presence of LLs	0.259	0.101	−0.17 (−0.37 to 0.03)
2b	Model 1+ total WML volume	0.276	<0.001	−0.03 (−0.04 to −0.01)
2c	Model 1+ presence of LLs and total WML volume	0.276	0.001	
3a	Model 1+ LL volume SLF	0.266	0.005	−1.46 (−2.48 to −0.45)
3b	Model 1+ LL volume ATR	0.267	0.003	−4.12 (−6.84 to −1.40)
3c	Model 1+ WML volume SLF	0.270	0.001	−0.13 (−0.21 to −0.05)
3d	Model 1+ WML volume ATR	0.273	<0.001	−0.23 (−0.35 to −0.10)
4a	Model 2c+LL volume SLF a	0.283	0.024	−1.20 (−2.24 to −0.16)
4b	Model 2c+LL volume ATR a	0.282	0.042	−3.12 (−6.13 to −0.11)
4c	Model 2c+WML volume SLF	0.276	0.570	0.06 (−0.14 to 0.25)
4d	Model 2c+WML volume ATR	0.276	0.826	−0.03 (−0.31 to 0.25)

The explained variance (R^2^) in executive functioning is given for each model with the corresponding p-value for the difference in explained variance (Δ R^2^) between the model and the previous model. Unstandardized coefficients (B) with corresponding 95% CIs are provided. LL: lacunar lesion. WML: white matter lesion. SLF: superior longitudinal fasciculus. ATR: anterior thalamic radiation. Presence of LLs (which corresponds with presence of LLs anywhere in the brain) was entered as a dichotomous variable; all other variables were entered as continuous variables.

a These results suggest that LLs in the anterior thalamic radiation (B = −3.12) might have greater impact on executive functioning than LLs in the superior longitudinal fasciculus (B = −1.20).

## Discussion

We found an association between the presence of white matter lesions in the superior longitudinal fasciculus and the anterior thalamic radiation and poor executive functioning using assumption-free voxel-based lesion-symptom mapping in a cohort of people with manifest arterial disease. Furthermore, in our region of interest-based linear regression model, we demonstrated a statistically significant association between the regional volume of lacunar lesions located in the superior longitudinal fasciculus and the anterior thalamic radiation and executive functioning after adjustment for total lacunar lesion and white matter lesion burden. This identifies these tracts as key anatomical structures in executive functioning and emphasizes the role of strategically located vascular lesions in vascular cognitive impairment.

Previous studies have demonstrated associations between LL and WML volume and poor cognitive functioning in patients with small vessel disease [5]–[8], [30]. Previously identified strategic locations include periventricular white matter [11], [31] and loci in the bilateral inferior frontal white matter [9]. WMLs and LLs in these loci were associated with poor executive functioning. However, these earlier studies did not use white matter tract-based analyses to study the relation between WMLs or LLs and cognition. The present study provides further insight into the relation between vascular lesion location and cognition by using both assumption-free VLSM and white matter tract-based analyses. Based on the VLSM results the anterior thalamic radiation and superior longitudinal fasciculus were selected to perform region of interest-based analyses. In these analyses we demonstrated a statistically significant association between LL volume within the anterior thalamic radiation and superior longitudinal fasciculus and executive functioning, independent of total WML and LL burden. These findings are in line with a recent VLSM study in patients with CADASIL, which is a genetically defined form of small vessel disease, in which an association was found between LLs and WML in the anterior thalamic radiation and the forceps minor and processing speed [10]. Ischemic lesions within these structures were a better predictor of processing speed than total lesion volume. Executive functioning was not assessed in the CADASIL study. In the current study no significant association was found between lesions in the anterior thalamic radiation and the forceps minor and processing speed using VLSM. There are three important explanations for this discrepancy in results. First, CADASIL is a monogenic disorder with a distinct pattern of lesion distribution affecting mostly the temporal lobes and external capsule [32]. Second, the current study was performed in patients with a relatively low total LL and WML burden compared to patients with CADASIL, although the lesion burden of patients with manifest arterial disease is higher than in the general population [33]. Third, most patients who were included in the SMART-MR study had normal cognition (table 2). This may explain why the total burden of small vessel disease explained only a small proportion of variance in cognition. Small vessel disease causes prominent early executive dysfunction, which might explain why in the current study an association was found between LLs and WMLs and executive functioning, but not speed or memory [34].

Our findings identify the anterior thalamic radiation and superior longitudinal fasciculus as key white matter tracts for executive functioning, which is in line with previous literature on the involvement of these structures in cognitive functioning. The anterior thalamic radiation consists of fibers between the anterior thalamic nuclei and the anterior cingulate cortices, and fibers connecting mediodorsal thalamic nuclei and the frontal cortex. [35]. These projections participate in prefrontal-subcortical circuits, which are known to be involved in executive functioning. [36], [37]. The superior longitudinal fasciculus is a major association pathway that connects frontal, parietal and temporal association areas [38]. It is known to play a central role in many higher brain functions, including executive functioning and intelligence [39].

The current study has some limitations. First, the lesion load in a large part of the brain did not exceed the pre-defined threshold (as illustrated by figure 2). Therefore, these brain regions could not be included in the VLSM analysis. Also, functionally relevant locations within analysed brain regions might have been missed because there were too few lesions. VLSM could not be performed for LLs because no single voxel was affected in at least five patients. Nevertheless, we were able to study the relation between the location of LLs and executive functioning using the region of interest-based analysis. Third, the SMART study population represents a selected patient cohort, on which additional exclusion criteria were applied for the purpose of the present study (i.e. patients with territorial infarcts, patients not suitable for cognitive testing). Moreover, patients with severe brain atrophy were more likely to be excluded from the study because of registration difficulties. This selection process, although inherent to the design of the present study, affects the generalizability of the findings. Finally, we cannot exclude errors associated with normalization and registration processes. However, we consider these errors to be small as all images were rigorously controlled by visual inspection.

In summary, the current study demonstrated an association between white matter lesions located in the superior longitudinal fasciculus and the anterior thalamic radiation and executive functioning in patients with manifest arterial disease using assumption-free voxel-based lesion-symptom mapping. Moreover, regional volume of lacunar lesions located in the superior longitudinal fasciculus and the anterior thalamic radiation was related to executive functioning, independent of total lacunar lesion and white matter lesion burden. These findings identify these white matter tracts as key anatomical structures in executive functioning and emphasize the role of strategically located vascular lesions in vascular cognitive impairment.
